# Methadone alters the peripheral inflammatory and central immune landscape following prenatal exposure in rats

**DOI:** 10.3389/adar.2022.10792

**Published:** 2022-11-29

**Authors:** Nethra K. Madurai, Yuma Kitase, Sarah Hamimi, Shannon E. Kirk, Riley Sevensky, Sindhu Ramachandra, Sankar Muthukumar, Vikram Vasan, Maide Ozen, Gwendolyn Gerner, Shenandoah Robinson, Lauren L. Jantzie

**Affiliations:** ^1^ Division of Neonatal-Perinatal Medicine, Department of Pediatrics, School of Medicine, Johns Hopkins University, Baltimore, MD, United States; ^2^ Division of Pediatric Neurosurgery, Department of Neurosurgery, School of Medicine, Johns Hopkins University, Baltimore, MD, United States; ^3^ Department of Neuropsychology, Kennedy Krieger Institute, Baltimore, MD, United States; ^4^ Department of Psychiatry and Behavioral Sciences, School of Medicine, Johns Hopkins University, Baltimore, MD, United States; ^5^ Department of Neurology and Developmental Medicine, Kennedy Krieger Institute, Baltimore, MD, United States; ^6^ Department of Neurology, School of Medicine, Johns Hopkins University, Baltimore, MD, United States

**Keywords:** methadone, inflammation, prenatal opioid exposure, immune priming, PBMC, SPIHR

## Abstract

Opioid use during pregnancy continues to rise at alarming rates with a parallel trend in the number of infants and children exposed to opioid medications each year. Prenatal opioid exposure (POE) occurs at a critical timepoint in neurodevelopment disrupting intricate pathways essential for neural-immune maturation with the potential for devastating long-term consequences. Understanding the mechanisms underlying injury associated with POE is essential to address long-term outcomes and identify diagnostic and therapeutic biomarkers in this vulnerable patient population. Using an established preclinical model of POE, we investigated changes in cerebral and peripheral inflammation and peripheral blood mononuclear cell (PBMC) activity. We hypothesized that neuroinflammation, as defined by changes in specific cerebral immune cell populations, would exist in adult rats following POE concomitant with sustained peripheral immune hyperreactivity (SPIHR). Our data demonstrated alterations in cerebral immune cells at postnatal day 60 (P60) typified by increased regulatory T cells (*p* < 0.01) and neutrophils (*p* < 0.05) in rats with POE compared to controls. Evaluation of serum revealed increased levels of IL-6 (*p* < 0.05) and CXCL1 (*p* < 0.05) at P21 in rats with POE compared to controls with no significant difference in cytokine or chemokine levels between the two groups at P60. Additionally, PBMCs isolated from rats with POE at P21 demonstrated baseline hypersecretion of IL-6 (*p* < 0.01) and SPIHR with increased levels of TNF-α (*p* < 0.05) and CXCL1 (*p* < 0.05) following stimulation with LPS. At P60, however, there was no significant difference found in cytokine or chemokine levels secreted by PBMCs isolated from rats with POE at baseline or with LPS stimulation when compared to controls. Taken together, these data demonstrate cerebral inflammation months after prenatal opioid exposure and long after the resolution of systemic inflammation and SPIHR seen at toddler age equivalent. Chronic alterations in the cerebral immune cell populations secondary to prenatal opioid exposure may underly long-term consequences of developmental brain injury including deficits in cognition and attention. These findings may be invaluable to further investigations of precise biomarkers of injury and targeted therapeutics for this vulnerable population.

## Introduction

The crisis of opioid use in the United States continues to grow and has a significant impact on many populations including pregnant women and children. Opioid use disorder during pregnancy has risen at alarming rates in the past decade with a 131% increase from 2010 to 2017 in women with a maternal opioid-related diagnosis at time of delivery (1). Paralleling this epidemic has been a sharp increase in the number of infants exposed *in utero* to opioid medications each year. Neonatal opioid withdrawal syndrome (NOWS) is a well-recognized consequence of prenatal opioid exposure (POE) in the first few weeks of life leading to an extended length of hospitalization for many newborn infants. However, the long-term adverse outcomes associated with POE are just beginning to be understood (2). As the epidemic grows, understanding the mechanisms underlying POE and the long-term consequence of this exposure is paramount to supporting the health and development of this vulnerable population.

Prenatal opioid exposure occurs as a result of maternal use or misuse of prescription opioid medications including oxycodone, hydrocodone, morphine, codeine and fentanyl, or illicit opioids, such as heroin. While it is known that POE is associated with an increased risk of fetal growth restriction and preterm birth, the effects of POE on the developing central nervous system remain poorly understood (3–5). Clinical data shows evidence of abnormal brain development with decreased brain volumes and aberrant structural connectivity in children exposed to opioid medications prenatally (6–12). Others have shown significant cognitive and motor dysfunction in school-age children with POE when compared to age-matched peers without prenatal opioid exposure (13). Larger cohort studies are limited by social, economic, and environmental confounding factors, but demonstrate higher risk of attention deficit hyperactivity disorder and symptoms in school-age children with prenatal opioid exposure as well (3, 14). Elegant preclinical studies using animals show similar findings and elicit concern for long-term neurobehavioral consequences in this patient population. Specifically, prenatal methadone exposure changes open field activity, and impairs sensorimotor developmental milestone acquisition concomitant with reduced neuronal density in motor cortex and aberrant circuit connectivity (15). Prenatal Methadone and buprenorphine cause impaired recognition memory, and nonspatial reference learning in young adult rats (16) corroborating reports of impaired cognitive flexibility and learning acquisition older adults after POE using a touchscreen platform (17). Similarly, buprenorphine and methadone impair social interaction and novel object recognition, diminish elevated plus maze performance, and induce anxiety (18). Studies using other opioids yield similar results. Prenatal fentanyl exposure alters sensory processing defined by multiple changes in synapses, regional changes in excitatory and inhibitory tone, and diminished dendritic arbor (19). Prescription opioids, including oxycodone, disrupt afferent regulation of dopamine activity in the ventral tegmental area during development suggesting disruption of the trajectory of mesolimbic circuity maturation and providing connection to common neuropsychological outcomes such as anxiety, attention, and depression (20).

Understanding the mechanism of opioid-induced neural injury and the lasting impact of this injury across the lifespan is key to identifying therapeutic targets and tailoring intervention to high-risk patients at crucial times in development. The ability to recognize patients at higher risk of long-term adverse outcomes from POE with precise biomarkers is also essential in guiding clinical management for diagnosis and treatment (21–23). Here, we defined alterations in the immune system following POE to test whether adults with POE had persistent brain inflammation. Specifically, we conducted investigations of serum inflammatory cytokine and chemokine profiles and evaluated peripheral immune hyper-reactivity from toddler age equivalent at P21 to an adult human age equivalent at P60. We also used multiparameter flow cytometry to interrogate cerebral and peripheral blood immune cell population dynamics in adulthood. We hypothesized that POE would induce neuroinflammation that could be detected in adulthood, defined by changes in infiltrating immune cells. We also predicted there would be sustained inflammation alongside disruption in the function and composition of the peripheral immune system.

## Methods

### Animals

Sprague-Dawley rat dams and litters were maintained in a temperature and humidity-controlled facility with food and water available *ad libitum*. A 12-h dark/light cycle was maintained for all animals with lights on at 0800 h. All experiments were performed in strict accordance with protocols approved by the Institutional Animal Care and Use Committee (IACUC) at the Johns Hopkins University School of Medicine. Protocols were developed and performed consistent with National Research Council and ARRIVE guidelines (24). Litter size was similar between methadone-exposed and saline-exposed litters. As previously published, (17) weights were significantly lower in methadone-exposed litters as compared to saline-exposed litters. For each experiment described, the data represents true n (individual rats). For every experiment and outcome measure, we used offspring from at least 4 different litters per condition to control for litter effects. Male and female offspring were used in every outcome measure, and in approximately equal numbers where possible and dictated by experimental endpoint.

### Methadone exposure

**FIGURE 1:**
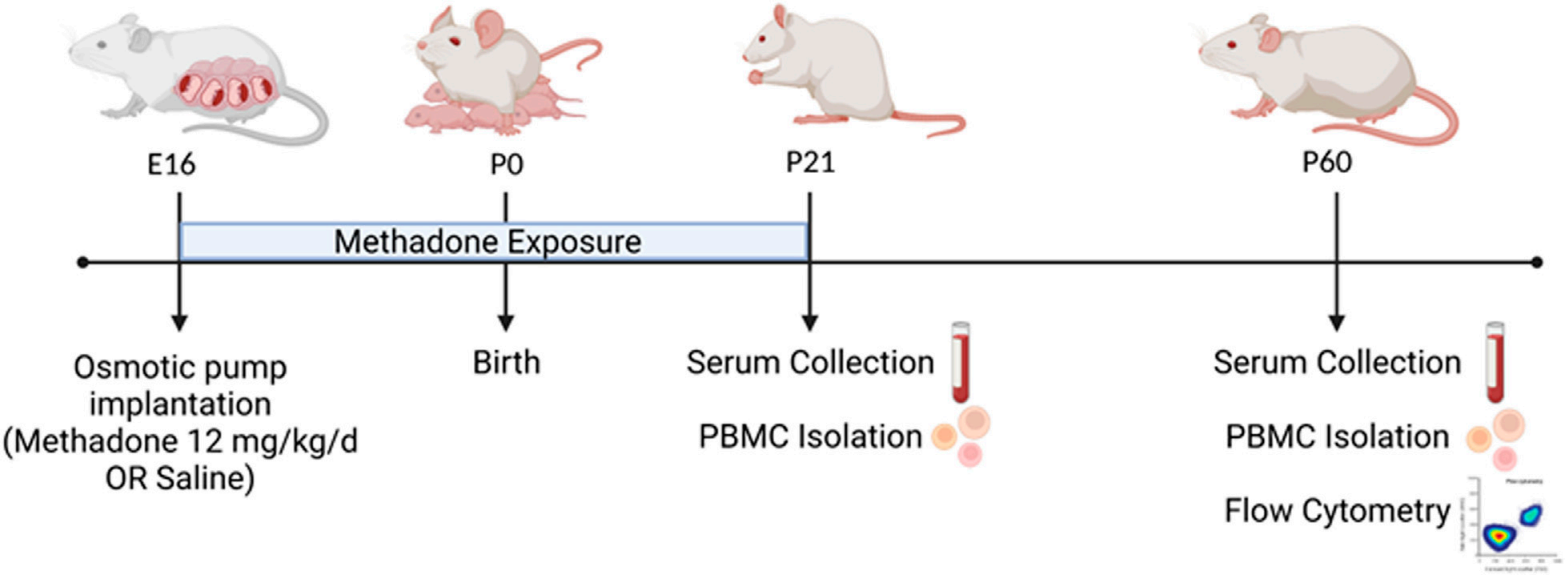
POE and Experimental Paradigm. Osmotic mini pumps were implanted into pregnant rat dams at embryonic day 16 (E16) for continuous methadone or saline exposure. Rat pups were born at term on E23/postnatal day 0 (P0) and continued to receive methadone or saline through maternal milk until weaning at P21. At P21 and P60, serum was collected and PBMCs isolated from all animals. At P60, brain and PBMCs were also collected for multiparameter flow cytometric analyses (Figure created with Biorender.com).

Per previously published methods, on embryonic day 16 (E16) osmotic mini pumps (ALZET, Cupertino, California) were implanted subcutaneously in the nape of the neck of pregnant rat dams for 28 days of continuous methadone (12 mg/kg, 0.25 μL/h flow rate) or sterile saline infusion (Figure 1) (17, 25). Methadone is a synthetic, long-acting, µ-opioid receptor agonist that readily crosses the placenta and blood-brain barrier. Specifically, following induction and maintenance of anesthesia with inhaled isoflurane, dams underwent minipump placement with a 1.5 cm transverse skin incision followed by careful blunt dissection of the subcutaneous space. Osmotic pumps were prefilled and primed prior to insertion, followed by closure of the space with sutures. The total duration of anesthesia was no longer than 7 min. Dams were carefully monitored following the procedure for full recovery. Rat pups were born at E23/postnatal day 0 (P0) following completion of gestation and remained with their dams. Pups continued to receive methadone or saline through the maternal milk supply until weaning on P21 (17, 25). Daily health checks were performed for pup wellbeing.

### Blood and brain collection

At P21 and P60, brain and blood were collected. Specifically, at the conclusion of each experiment, rats were deeply anesthetized, and venous blood was collected from the right atrium in pyrogen-free, K2 EDTA, vacutainers (BD Vacutainer, Franklin Lakes, NJ). Whole blood was then aliquoted as dictated by endpoint assays to further undergo PBMC isolation or serum separation. Brains from each animal were harvested at the time of blood collection.

### Flow cytometry

**FIGURE 2:**
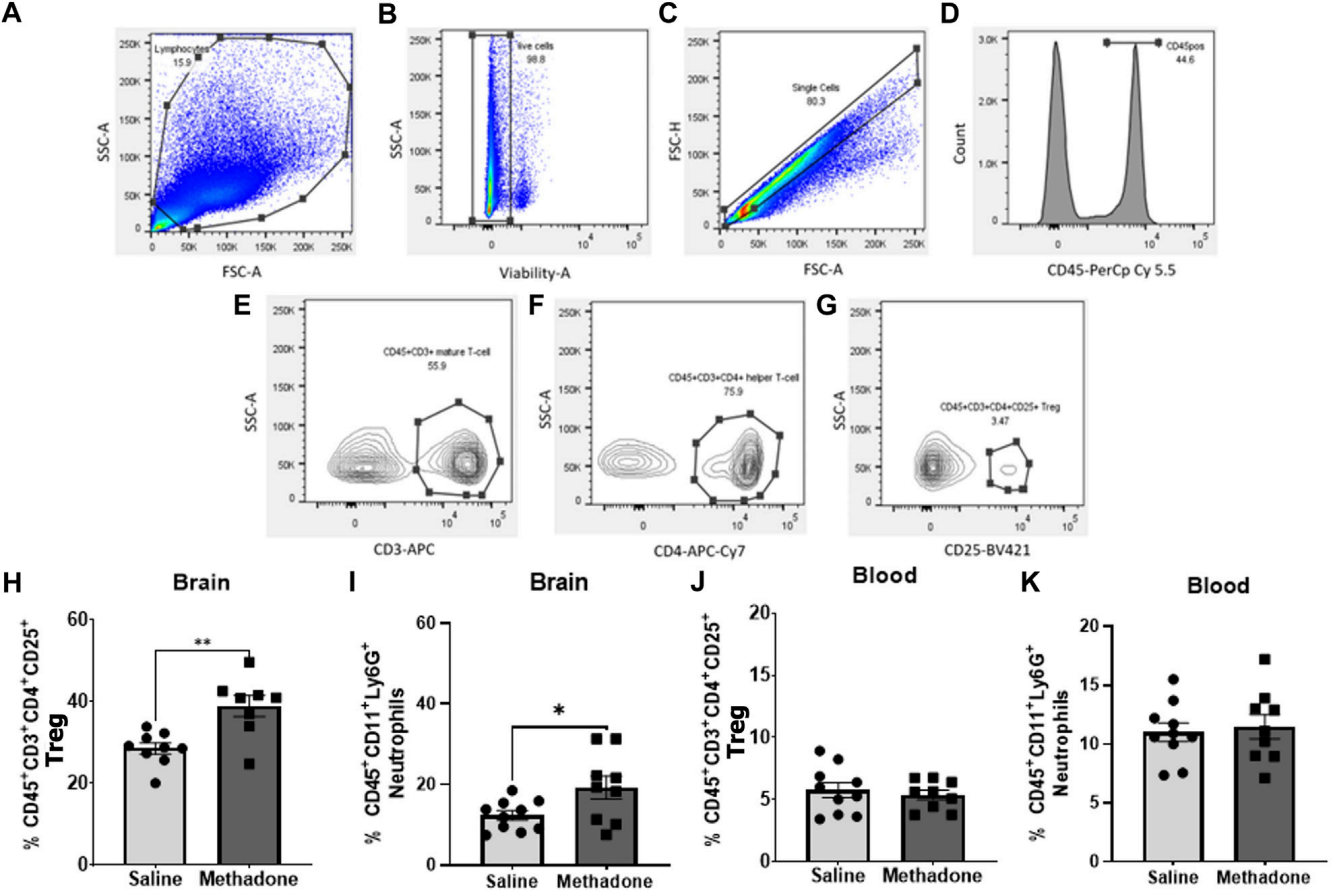
Adult rats have significantly altered central immune cell population dynamics following POE. Multiparameter flow cytometric analyses were used to identify changes in lymphocyte populations in blood and brain isolated from rats with POE and controls at P60. Lymphocytes were identified by size, granularity, and viability dye staining **(A–C)**. Further gating for CD45 **(D)**, CD3 **(E)**, CD4 **(F)** and CD25 **(G)** is shown. Increased regulatory T cells **(H)** and neutrophils **(I)** were observed in the brains of rats with POE compared to controls. No significant differences were found in regulatory T cells and neutrophils **(J,K)** in PBMCs isolated from rats with POE and controls (Mann-Whitney U-test for all, *p < 0.05, **p < 0.01, FSC, forward scatter; SSC, side scatter).

At P60, brain and peripheral blood mononuclear cells (PBMCs) were collected from adult rats for flow cytometry consistent previous reports (17, 25–31). Using a Miltenyi adult brain dissociation kit and Miltenyi gentleMACS™ protocol, whole cerebrum was digested for single-cell suspension (22–24). This suspension was passed through 70-µm cell filters and then underwent debris elimination using debris removal solution. Miltenyi red blood cell lysis solution (1x) was then used for complete removal of erythrocyte populations. Live cells were counted on a Countess™ II Automated Cell Counter (Thermo Fisher Scientific). Next, 1x10^6^ live cells were incubated with a saturating solution of Fc block (Clone D34-485, BD Biosciences, San Jose, CA) followed by staining with fluorochrome-conjugated viability dye and antibodies against: CD45-PerCp Cy5.5 or CD45-APC Cy7 (Clone OX1; eBiosciences, Waltham, MA), CD11b/c BV605 (Clone OX42; eBiosciences, Waltham, MD), Ly6G-FITC (Clone RB6-8C5, AbCam, Cambridge, MA), CD3-APC (Clone 1F4; BD Biosciences, San Jose, CA), CD4-APC Cy7 (W3/25; Biolegend, San Diego, CA), and CD25-BV421 (OX-39; BD Biosciences, San Jose, CA). Data were acquired using a BD LSR-II flow cytometer (BD Biosciences, San Jose, CA) and analyzed using FlowJo software v.10.7.1 (FlowJo LLC, Ashland, OR). Cells were first gated based on size and granularity (forward scatter (FSC) vs. side scatter (SSC)), followed by gating on live cells confirmed with viability dye staining. Single cells were then identified using FSC-A vs FSC-H. CD-45^+^ cells were identified and further analyzed for CD 11b/c expression. CD45^+^CD11b/c^+^ cells could then be gated for Ly6G expression to identify neutrophils (27, 32, 33). In the brain, CD45^+^ cells were further characterized. CD45^high^CD11b/c^+^ cells were considered macrophages and CD45^low/med^CD11b/c^+^ cells were considered resident microglia (23, 27, 28). Using a separate panel, T cells were identified by assessing CD45^+^CD3^+^ cells. CD45^+^CD3^+^CD4^+^ were classified as helper T cells and CD45^+^CD3^+^CD4^+^CD25^+^ as regulatory T cells (Tregs) (22, 23, 29, 30) (Figures 2A–G).

### Serum collection

Consistent with published methods, whole blood at P21 AND P60 was centrifuged at 6000 x g for 15 min at 4°C (17, 27, 32–36). Serum was then removed and stored at −80°C until cytokine and chemokine analysis. Repeated freeze-thaw cycles were avoided.

### Peripheral blood mononuclear cell isolation

PBMCs were isolated from saline-exposed and methadone-exposed rats using a Ficoll-gradient separation consistent with previously published methods (17, 25–27). In sterile 15-ml conical tubes, equal volumes of venous blood and RPMI (Roswell Park Media Institute) 1640 media (Gibco, Waltham, MA, United States) were placed in a layer on top of Ficoll-Plaque Plus (GE Healthcare, Chicago, IL, United States) and centrifuged at 400 x g for 30 min at room temperature. The PBMC cell layer was then collected and transferred into a new 15 ml conical tube and resuspended in RPMI media. Two wash cycles with RPMI media were performed by centrifuging the sample at 400 x g for 10 min at room temperature, followed by supernatant disposal and resuspension of the pellet. Following the wash cycles, the PBMC cell pellet was resuspended in media and a cell density of 1 × 10^6^ cells/mL per well was plated in duplicate on 3.5 cm Petri dishes.

### Peripheral blood mononuclear cell treatment with LPS

PBMCs from saline-exposed and methadone-exposed rats were plated and treated with media only or stimulated with LPS at a concentration of 100 ng/ml (17, 25–27). Media and cells were collected at 3 h after stimulation and 24 h after stimulation to assess PBMC secretory activity and changes prior to and after protein synthesis. Cells and supernatant were stored in sterile tubes at −80°C until further analysis. Each culture, condition and exposure were performed in duplicate. Repeat freeze-thaw cycles were avoided.

### Multiplex electrochemiluminescent immunoassay

Cytokines and chemokines in serum samples and supernatant from cultured PBMCs (secretome) were analyzed using a V-PLEX Proinflammatory Panel 2 Rat Kit (K15059D; Meso Scale Diagnostics, Rockville, MD, United States) (17, 18, 22, 23, 25, 26, 31, 32). The following cytokine and chemokine secretions were assessed: interferon gamma (IFN-γ), interleukin-1β (IL-1 β), IL-4, IL-5, IL-6, IL-10, IL-13, chemokine (C-X-C motif) ligand 1 (CXCL1) and tumor necrosis factor-α (TNF-α). The assay was performed according to manufacturer specifications. Each sample of PBMC culture media and serum was diluted 1:3 and loaded in duplicate with prepared standards onto blocked and washed 96-well plates. Following a series of washes and incubation with antibody detection solution, plates were washed and loaded with read buffer onto a Quickplex SQ 120 Imager (25–27, 37–40). Consistent with the standard in the field, samples reading below the detectable limit of the assay or with a coefficient of variation greater than 25% in an individual assay were removed from further analysis (25–27, 37–40). The V-PLEX pro-inflammatory panel assay is performed with less than 10% variability between runs and sensitivity in sub pg/mL ranges.

### Statistical analysis

Data are represented as mean ± the standard error of the mean (SEM). Data was tested for normality using the Shapiro-Wilk test. Statistical differences between 2 groups of parametric data were established with Student’s *t*-test and non-parametric data with the Mann-Whitney test with *p* < 0.05 considered statistically significant. GraphPad Prism 9.3.1 software was used to perform statistical analyses.

## Results

### Prenatal opioid exposure induces changes in central immune cell populations

At P60, using multiparameter flow cytometric analyses, PBMCs and brain immune cell populations were examined in-depth for rats with POE versus controls (*n* = 10/group (5 males; 5 females) for saline, *n* = 9/group (5 males; 4 females) for methadone). Flow cytometric analyses of the brain revealed immune cell population changes following POE, with a significant increase in regulatory T cell (CD45^+^CD3^+^CD4^+^CD25^+^) populations in rats with POE as compared to controls (saline: 28.57 ± 1.35%, methadone: 38.95 ± 2.58%, mann whitney U-test, *p* < 0.01) (Figure 2H). Furthermore, there was a significant increase in CD45^+^CD11b/c^+^Ly6G^+^ neutrophils in the brains of rats with POE as compared to controls (saline: 12.33 ± 1.18%, methadone 19.24 ± 2.86%, mann whitney U-test, *p* < 0.05) (Figure 2I). There was no significant difference in helper T cell (CD45^+^CD3^+^CD4^+^) populations, resident microglia (CD45^low/med^CD11b/c^+^) or infiltrating macrophages (CD45^high^CD11b/c^+^) in the brain (data not shown). Flow cytometric analyses of immune cells in the blood revealed no significant differences in helper T cells, regulatory T cells, infiltrating macrophages, or neutrophils (Figures 2J,K). Overall, these data demonstrated cerebral inflammation at P60 defined by increased regulatory T cells and neutrophils in rats with POE.

### Peripheral inflammation from POE is evident beyond the neonatal period

**FIGURE 3:**
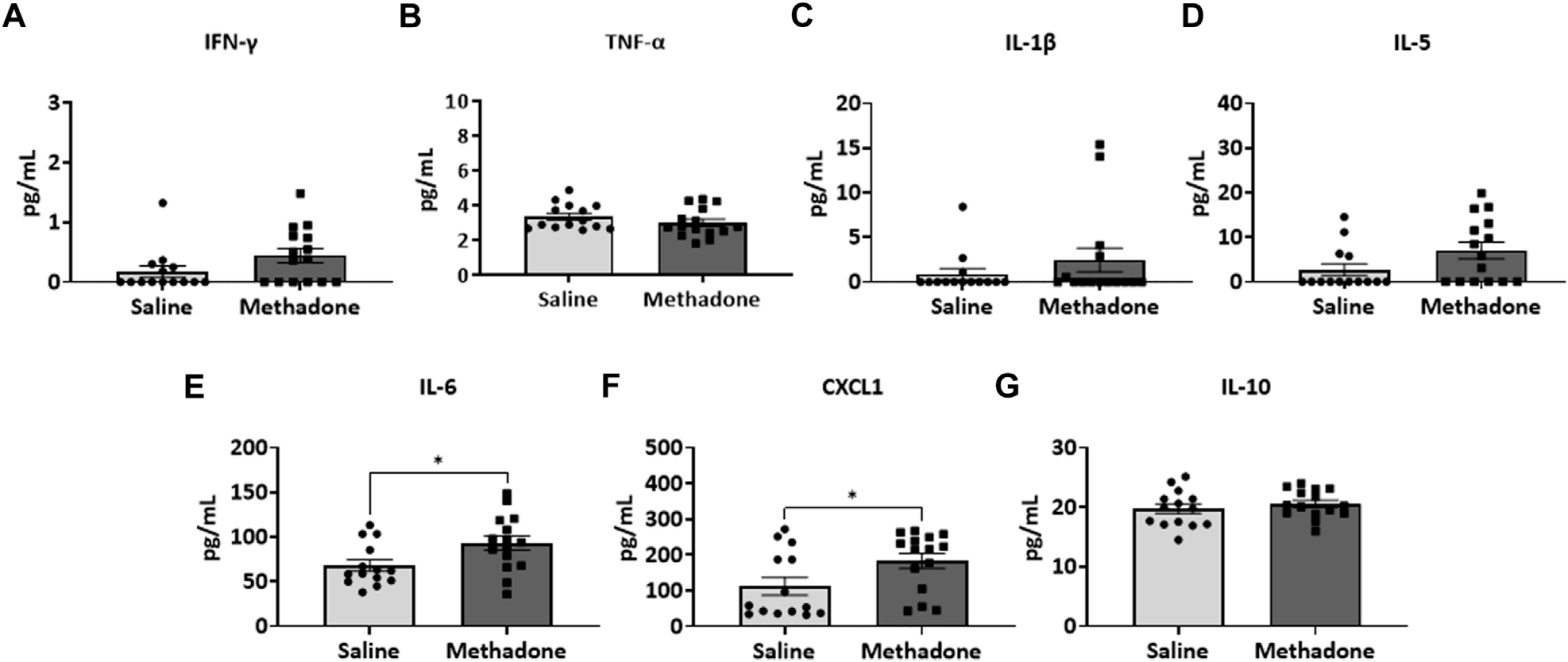
POE leads to elevations in serum inflammatory cytokines and chemokines at P21. Osmotic mini pumps with methadone or saline were implanted in pregnant dams at E16. Pups were born and serum was collected at P21 and assayed using a translatable multiplex electrochemiluminescent biomarker platform. At P21, methadone-exposed rats demonstrate significantly elevated levels of IL-6 **(E)** and CXCL1 **(F)** compared to controls reflecting persistent peripheral inflammation following POE at a toddler age equivalent. (t-test for all, *p < 0.05).

**FIGURE 4:**
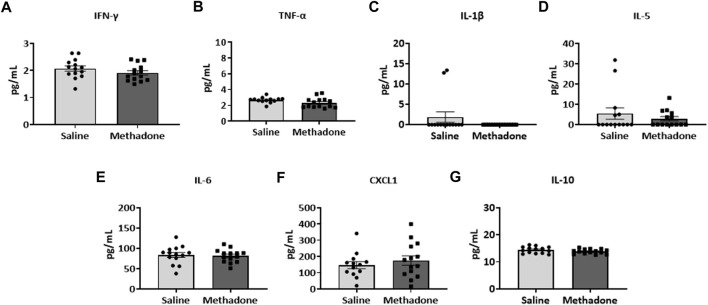
Peripheral inflammation secondary to prenatal opioid exposure normalizes by P60. Osmotic minipumps primed with methadone or saline were implanted in pregnant dams at E16. Pups were born and serum was collected at P60 and assayed using a translatable multiplex electrochemiluminescent biomarker platform. At P60, no significant differences were observed between methadone-exposed and saline-exposed rats (t-test for all).

Detailed investigation of inflammatory markers in serum was undertaken at both P21 and P60 to establish potential differences in secreted proteins with methadone exposure and inflammatory network activation. Analysis of the serum following POE began with measurement of cytokine and chemokine levels at P21, approximately human toddler age equivalent (*n* = 15/group; 7 males; 8 females) (33). Rats with POE had a significant elevation in IL-6 (saline: 68.11 ± 6.34 pg/ml, methadone: 93.08 ± 8.11 pg/ml, t-test, *p* < 0.05) (Figure 3E) and CXCL1 (saline: 111.9 ± 24.8 pg/ml, methadone: 183.6 ± 21.2 pg/ml, t-test, *p* < 0.05) compared to controls (Figure 3F). At P60, an adult equivalent age (23), evaluation of serum cytokine and chemokines revealed no statistically significant difference in levels of IFN-γ, TNF-α, IL-1β, IL-5, IL-6, CXCL1, and IL-10 when comparing rats with POE to controls (*n* = 14/group; 7 males and 7 females) (Figure 4). In summary, this data shows increases in pro-inflammatory serum biomarkers at P21 following POE, defined by significant elevation of IL-6 and CXCL1 in rats with POE, and normalization of serum inflammatory markers at P60.

### POE alters the baseline PBMC secretome

**FIGURE 5:**
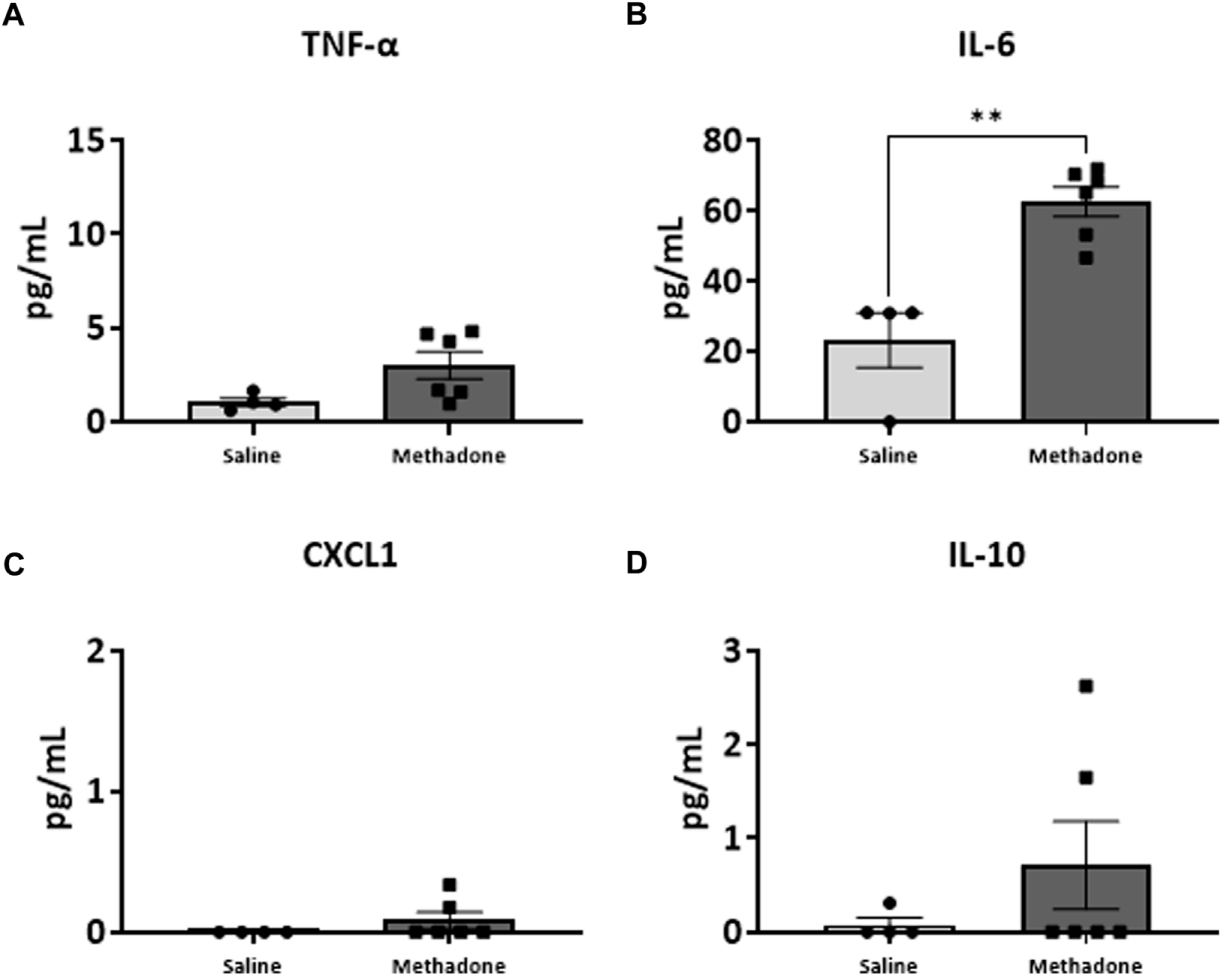
POE causes alterations in the PBMC secretome at baseline. PBMCs were isolated from P21 pups prenatally exposed to saline or methadone. Conditioned media was then assessed for cytokines and chemokines after 3 h in culture. PBMC secretome from rats with POE demonstrated significant elevation in IL-6 compared to controls. There was no significant difference in other cytokine and chemokine levels between the two groups (t-test for all, **p < 0.01).

**FIGURE 6:**
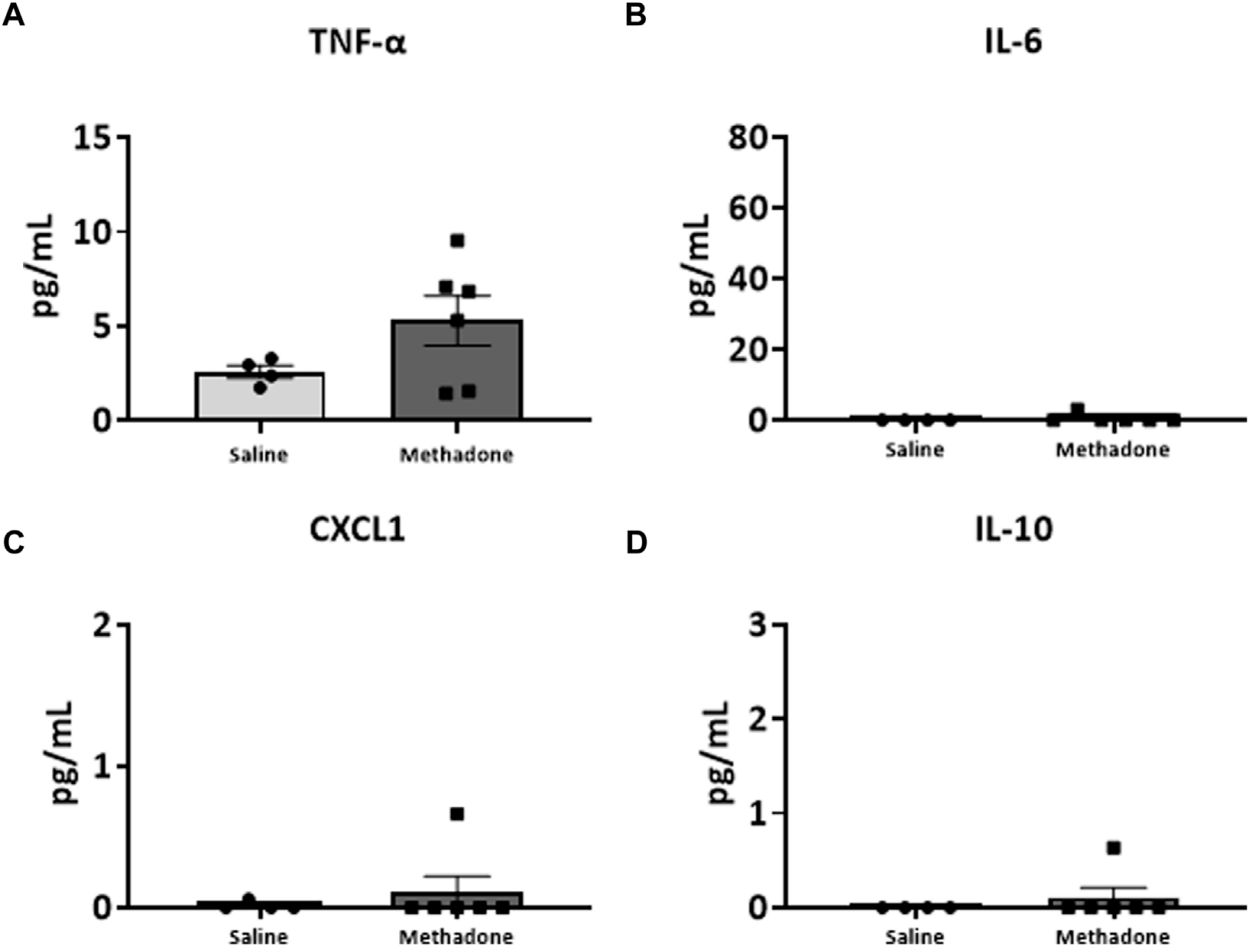
Alterations in baseline PBMC secretome are not observed after 24 h in culture. PBMCs were isolated from P21 pups prenatally exposed to saline or methadone. Conditioned media was then assessed for cytokines and chemokines after 24 h in culture. There were no significant differences in cytokine and chemokine levels between rats with POE and control animals (t-test for all).

Following our assessment of peripheral serum cytokine and chemokine levels, we further investigated peripheral immune system reactivity with evaluation of the baseline and stimulated PBMCs secretome at P21 (*n* = 4/group for saline; 2 males; 2 females, *n* = 6/group; 3 males; 3 females for methadone). At baseline, the conditioned media of PBMCs isolated from rats with POE demonstrated elevated levels of TNF-α levels at baseline in rats with POE after 3 h in culture (saline: 1.051 ± 0.220 pg/ml, methadone: 3.009 ± 0.721 pg/ml, *t*-test, *p* = 0.0655) although this failed to reach statistical significance (Figure 5A). IL-6 was significantly elevated at baseline compared to control rats (saline: 23.22 ± 7.74 pg/ml, methadone: 62.56 ± 4.19 pg/ml, t-test, *p* < 0.01) (Figure 5B). No other statistically significant differences were noted in baseline secretion of cytokines from PBMCs in culture after 3 h (Figures 5C,D) or 24 h (Figure 6) in culture, including CXCL1, IL-10, IFN-γ, IL-5, and IL-1β. Together, these results indicate that the PBMC secretome at baseline in P21 rats with POE is altered and favors a pro-inflammatory microenvironment.

### POE induces sustained peripheral immune hyper-reactivity (SPIHR)

**FIGURE 7:**
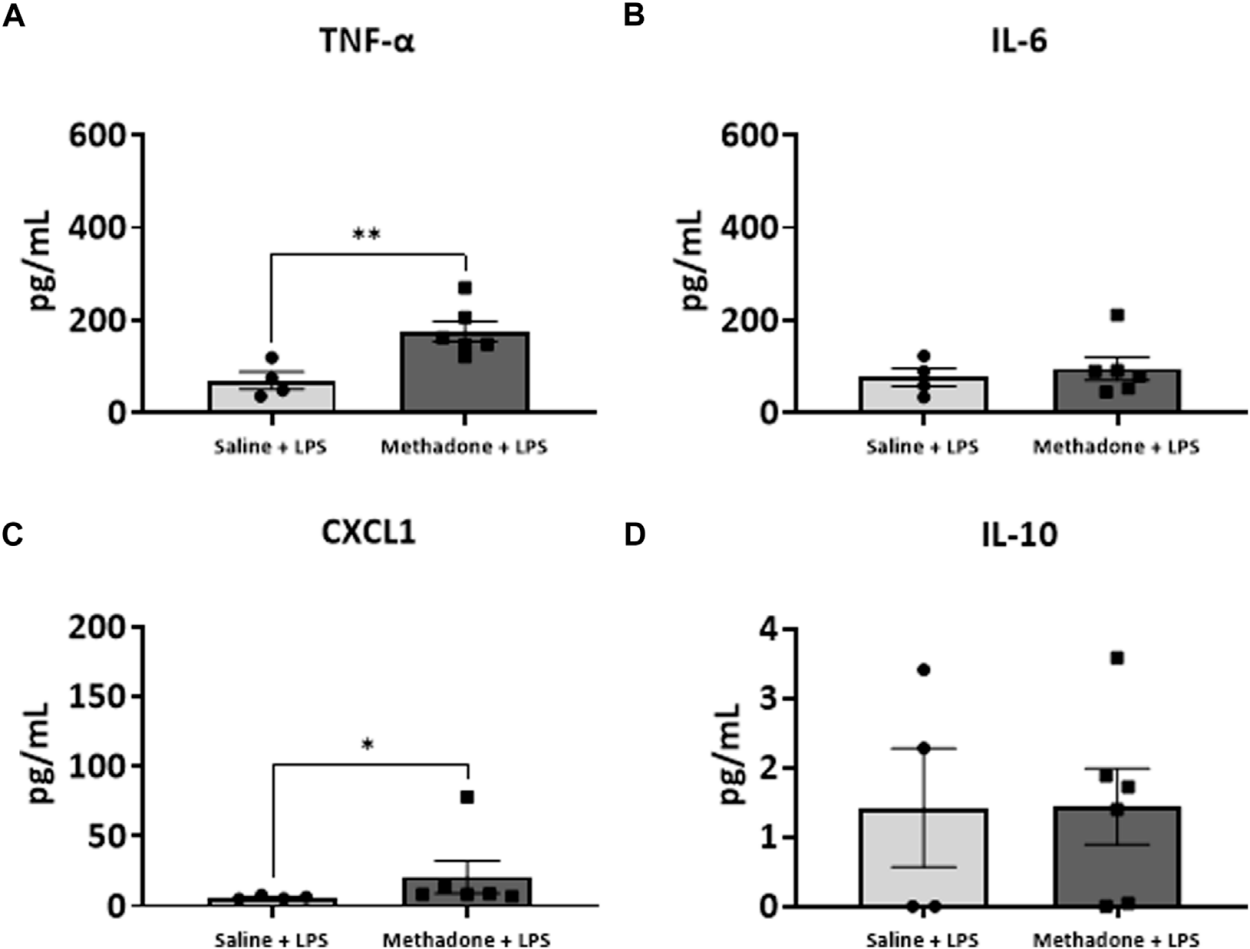
POE induces sustained peripheral immune hyper-reactivity (SPIHR) after 3 h in culture. PBMCs were isolated from P21 with POE. Conditioned media was then assessed for cytokine and chemokine levels after PBMCs were in culture for 3 h and following challenge with LPS. PBMC secretome from rats with POE demonstrated significant elevation in TNF-α levels compared to controls when stimulated with LPS (t-test for all, *p < 0.05, **p < 0.01).

**FIGURE 8:**
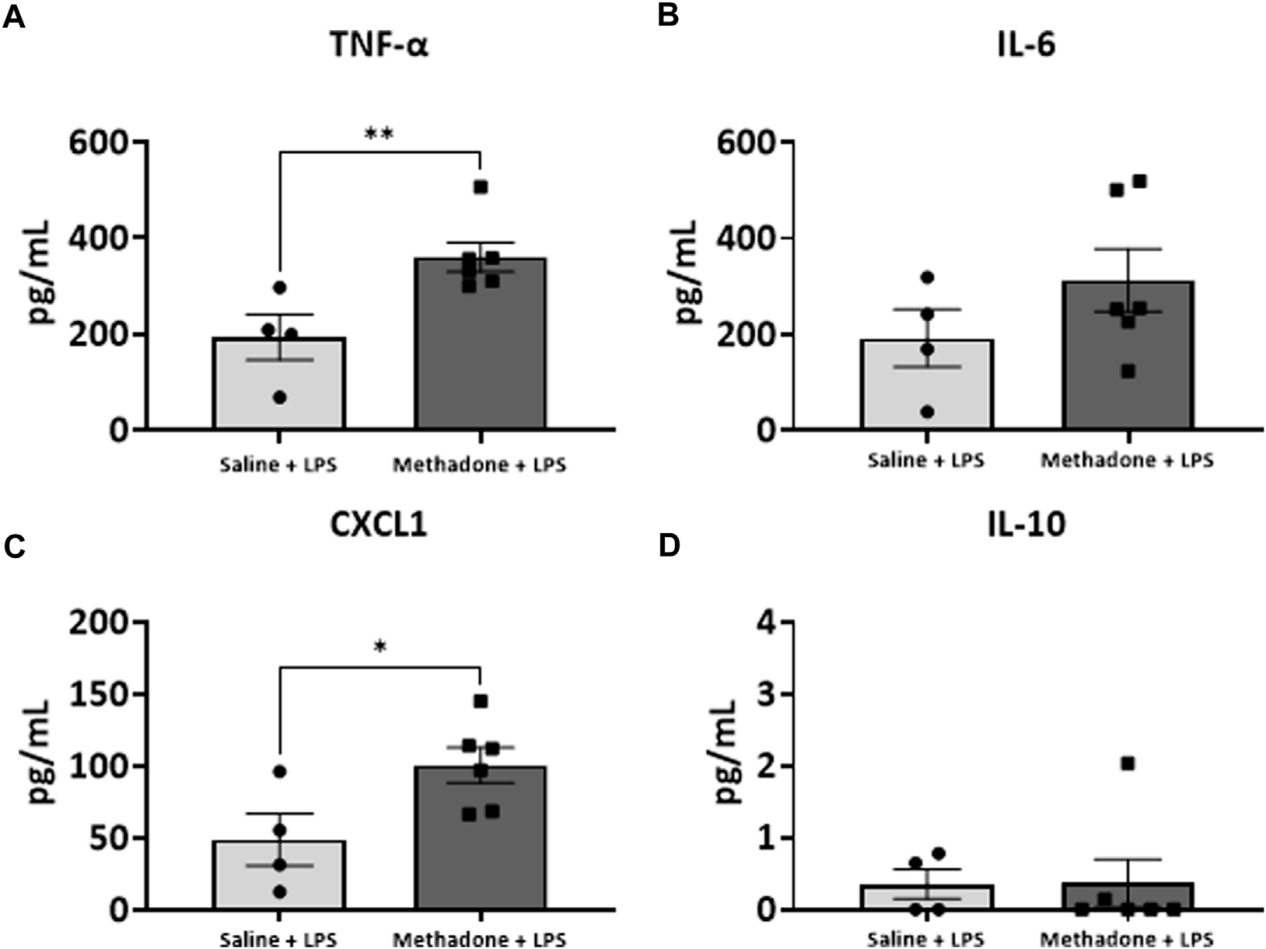
POE induces SPIHR after 24 h in culture. PBMCs were isolated from P21 pups. Conditioned media was then assessed for cytokine and chemokine levels after 24 h in culture following challenge with LPS. PBMC secretome from rats with POE demonstrated significant elevation in TNF-α and CXCL1 levels compared to controls (*p < 0.05, **p < 0.01).

We next assessed the reactivity of PBMCs following a secondary immune challenge performed with LPS (*n* = 4/group for saline; 2 males; 2 females, *n* = 6/group; 3 males; 3 females for methadone). This was necessary to unmask characteristics of PBMC responsiveness following a secondary insult. There were significant elevations in TNF-α levels in PBMCs isolated from P21 rats with POE after LPS challenge at both 3h and 24 h in culture. Specifically, there was a notable 2.5-fold increase in TNF-α levels in rats with POE compared to controls following LPS stimulation at 3 h (saline: 69.68 ± 18.42 pg/ml, methadone: 175.9 ± 22.0 pg/ml, t-test, *p* < 0.01) (Figure 7A). There was also a more than three-fold increase in CXCL1 levels in rats with POE compared to controls after 3 h in culture with LPS (saline: 6.226 ± 0.65 pg/ml, methadone: 20.68 ± 11.6 pg/ml, t-test, *p* < 0.05) (Figure 7C). These increases in TNF-α and CXCL1 levels in the PBMC secretome of rats with POE was also seen after 24 h in culture with an almost 2-fold increase in TNF-α levels (saline: 193.4 ± 47.2 pg/ml, methadone: 360.6 ± 30.8 pg/ml, t-test, *p* < 0.01) (Figure 8A), and a 2-fold increase in CXCL1 levels (saline: 48.89 ± 18.07 pg/ml, methadone: 100.7 ± 12.3 pg/ml, t-test, *p* < 0.05) (Figure 8C). Following secondary immune challenge with LPS, the PBMC secretome in rats with POE demonstrates SPIHR with significant elevation in TNF-α and CXCL1 after both 3 and 24 h in culture.

### Peripheral immune reactivity and hypercytokinemia normalize by P60

After assessing peripheral immune reactivity and the inflammatory profile at P21, we expanded our assessment by investigating the PBMC secretome at baseline and with LPS challenge from rats with POE and controls at P60 (*n* = 5/group for saline (2 males; 3 females), *n* = 7/group (4 males; 3 females) for methadone). Overall, individual cytokine and chemokine expression levels were lower than the levels noted at earlier timepoints and developmental stages. There were no significant differences in any of the cytokine or chemokine levels including TNF-α, IL-6, CXCL1, IL-10, IFN-γ, IL-5, and IL-1β at baseline between POE and controls after both 3 and 24 h in culture (*p* > 0.05 for all, t-tests, data not shown). Following stimulation with LPS, cytokine and chemokine levels increased but again remained similar between the methadone-exposed and saline-exposed groups with no significant differences found after both 3 and 24 h in culture (*p* > 0.05 for all, t-tests, data not shown).

## Discussion

Given the continued rise in opioid use across the globe, there is an urgent need to address the evolving public health crisis affecting countless pregnant women and children exposed to opioid medications *in utero*. Alongside growing recognition of the long-term neurologic impact associated with *in utero* opioid exposure, an improved understanding of the mechanisms underlying the brain injury caused by POE is imperative to establish biomarkers for identification of injury and to determine novel therapeutic targets. POE occurs at a critical timepoint in development disrupting delicate pathways essential for proper maturation of neural-immune function. Opioids readily cross the placenta and blood-brain barrier and lead to direct stimulation of inflammatory pathways *via* TLR4-mediated signaling (41–43). By shifting these pathways towards a pro-inflammatory state, opioids alter the developing immune system, and this alteration is sustained throughout the lifespan (2, 17, 44).

In this study, we show shifts in cerebral immune cell populations, defined specifically by increased neutrophils and regulatory T-cells, occurring months after prenatal opioid exposure. Furthermore, we demonstrate evidence of peripheral inflammation alongside immune priming and sustained peripheral immune reactivity (SPIHR) following prenatal opioid exposure that extends beyond the neonatal period. Even as markers of serum inflammation and SPIHR normalize into adulthood, elevated cerebral neutrophil and regulatory T-cell levels remain, highlighting the long-term impact of prenatal opioid exposure on the brain. Understanding the precise mechanisms underlying this injury is crucial to identifying those children at high risk of injury and to identifying targets for neuroimmunomodulation.

Previously, using the same model of POE, we identified a robust systemic inflammatory response syndrome and immune system dysfunction during the neonatal period concomitant with microstructural white matter injury and cognitive deficits in adulthood (17). POE led to priming of the immune system in the immediate perinatal period with significant baseline elevation in secretion of pro-inflammatory cytokines and chemokines, as well as an exaggerated inflammatory response from PBMCs acutely after stimulation with LPS (25). Specifically, we found of significant elevation of the inflammatory cytokines IL-1β, TNF-α, IL-6, and CXCL1 in the peripheral circulation at P10, around human term age equivalent, in POE rats suggesting a systemic inflammatory response syndrome (SIRS)-like response. We also showed that PBMCs, at P7, demonstrate significant baseline hypersecretion of TNF-α, CXCL1, and IL-6 with decreased levels of anti-inflammatory interleukin-10 (IL-10) and an exaggerated response to LPS stimulation with increased levels of TNF-α, CXCL1, IL-6, and IL-10 in rats with POE compared to controls (17, 25). Here, we extend those finding to show increased serum CXCL1 and IL-6 at P21, in POE rats concomitant with pro-inflammatory, dynamic, PBMC reactivity at P21, toddler age equivalent. CXCL1 is a potent chemokine responsible for neutrophil chemotaxis that has been implicated in significant intrauterine, placental, and fetal inflammation secondary to chorioamnionitis (26, 27, 33). Dysregulation in peripheral cytokine levels is not unique to POE and has also been identified in children with neonatal encephalopathy, cerebral palsy, and trisomy 21 (45–48). Our PBMC data reveals baseline hypersecretion of IL-6 that persists at P21. Further interrogation of PBMCs in culture with LPS stimulation at P21 demonstrates dysregulation in immune response following POE. After a 3 and 24 h incubation period, we note hypersecretion of TNF-α and CXCL1 in methadone-exposed PBMCs compared to saline-exposed PBMCs. These alterations in the baseline immune system function and immune response are indicative of SPIHR. The implications of inflammation during this timeframe from the neonatal period into toddler age equivalent cannot be understated, as it coincides with the elaborate neurodevelopmental program guiding myelination, oligodendrocyte maturation, and neural circuit formation that remains vulnerable to disruption (49, 50). Taken together with emerging clinical literature supporting priming of the immune system secondary to opioid exposure *in utero*, these data support a unique inflammatory signature followed by increased sensitivity to future insults. This altered immune landscape may lead to aberrant neural maturation and long-term cognitive and functional impairment that is just starting to be recognized in countless children and adults who have been exposed to opioid medications prenatally and has been demonstrated in elegant preclinical studies (15, 19–23).This suggests important future directions to identify correlations with severity of brain injury with functional magnetic resonance imaging, longitudinal assessment of white matter injury, and assessment of additional domains of cognition, including attention and inhibitory control using the touchscreen platform.

To our knowledge, this is the first investigation highlighting changes in cerebral immune cell populations into adulthood following opioid exposure commencing *in utero*. Our data shows persistently abnormal populations of cerebral lymphocytes following POE, with increased regulatory T cells and neutrophils compared to control rats. This finding offers invaluable insight into long-term immune alterations that coincides with long-term deficits seen in individuals with prenatal opioid exposure (51–53). It also underscores the need for improved biomarker development to better identify those at risk of persistent inflammation into adulthood including imaging or functional assessments of injury. Furthermore, increased regulatory T cells in the brain is consistent with other models of perinatal brain injury that show tissue injury, neuronal loss, and abnormal long-term neurodevelopment associated with increases in T cells (54). Indeed, modulation of regulatory T cells may be neuroprotective under specific conditions of developmental brain injury and offers an important therapeutic avenue for neuroimmunomodulation to treat brain injury and neuroinflammation secondary to POE (54). Serum elevations of IL-6 and CXCL1, as well as hypersecretion of TNF-α and CXCL1 following LPS stimulation of PBMCs in rats with POE at P21 may also serve as valuable markers of earlier dysfunction in the toddler age equivalent that could suggest risk for future persistent immune alterations. This is evident as CXCL1, a potent chemokine in neutrophil chemotaxis, is upregulated after POE and exerts a lasting impact on cerebral inflammation with a persistent increase of neutrophils months after opioid exposure. Promoting homeostasis, rather than antagonism of these unbalanced inflammatory pathways, may be crucial in the treatment of brain injury secondary to POE due to key neurodevelopmental roles of both CXCL1 and TNF-α.

Critically, our data in rats with POE also suggests that the period in which to intervene, from a clinical perspective, extends beyond the neonatal period. While ongoing inflammation, detected even months after cessation of exposure to opioids, can negatively impact brain function into adulthood (44), it also potentially broadens the timeframe for intervention. Targeting persistent inflammation into toddlerhood may still influence long-term developmental outcomes in this high-risk population. Further investigation of changes in immune cell populations in the peripheral circulation using flow cytometric analyses across a similar time course beginning at P21 may offer insight into the specific pattern of inflammation and immune dysregulation associated with POE and provide guidance for discovery of earlier biomarkers, especially in discrete developmental subsets. Additionally, analysis of central immune cell populations in animals, including inflammatory activation and morphology assessments, may help characterize the impact of these alterations on the developing and aging CNS.

There are limitations to the design and scope of our study and future investigations will address these constraints. Firstly, our study was not powered to evaluate differences in outcome measures based on sex. Further investigation into sex as a modifier of inflammation secondary to POE is important to identifying at-risk infants and children and evaluating responsiveness to novel therapeutic approaches including neuroimmunomodulation. Second, opioid exposure in this model occurs from E16 through P21 and does not capture opioid exposure from the onset of pregnancy (E0). Last, a brief period of isoflurane anesthesia was administered during the third trimester of rat gestation to implant osmotic minipumps, and this may have been an additional inflammatory stimulus during pregnancy. Future studies will address the correlation between immune function, functional and structural brain injury, and deficits of cognition and attention in adulthood. Longitudinal assessment of inflammation and the immune system following POE in the same rat, over time would be beneficial.

In conclusion, we provide evidence of peripheral inflammation alongside immune hyper-reactivity following prenatal opioid exposure. The importance of neural-immune communication and crosstalk with the peripheral immune system and central immune system is further highlighted with durable changes in cerebral immune cell populations of regulatory T cells and neutrophils months after POE. Beyond molecular inflammation, this study demonstrates immune cell population changes in adulthood secondary to prenatal exposure that may be critical to understanding the underpinnings of injury associated with opioid exposure. This study adds to a growing body of important literature linking sustained changes in immune reactivity with developmental brain injury (45–48). Furthermore, this study offers insight into potential treatment targets and widens the time course for potential intervention to help countless children with brain injury resulting from prenatal opioid exposure.

## Data Availability

The raw data supporting the conclusion of this article will be made available by the authors, without undue reservation.
