# Corrigendum: Methadone alters the peripheral inflammatory and central immune landscape following prenatal exposure in rats

**DOI:** 10.3389/adar.2023.11272

**Published:** 2023-02-21

**Authors:** Nethra K. Madurai, Yuma Kitase, Sarah Hamimi, Shannon E. Kirk, Riley Sevensky, Sindhu Ramachandra, Sankar Muthukumar, Vikram Vasan, Maide Ozen, Gwendolyn Gerner, Shenandoah Robinson, Lauren L. Jantzie

**Affiliations:** ^1^ Division of Neonatal-Perinatal Medicine, Department of Pediatrics, School of Medicine, Johns Hopkins University, Baltimore, MD, United States; ^2^ Division of Pediatric Neurosurgery, Department of Neurosurgery, School of Medicine, Johns Hopkins University, Baltimore, MD, United States; ^3^ Department of Neuropsychology, Kennedy Krieger Institute, Baltimore, MD, United States; ^4^ Department of Psychiatry and Behavioral Sciences, School of Medicine, Johns Hopkins University, Baltimore, MD, United States; ^5^ Department of Neurology and Developmental Medicine, Kennedy Krieger Institute, Baltimore, MD, United States; ^6^ Department of Neurology, School of Medicine, Johns Hopkins University, Baltimore, MD, United States

**Keywords:** methadone, inflammation, prenatal opioid exposure, immune priming, PBMC, SPIHR

In the original article, there was an error in the **Funding** statement. The correct number for the National Institutes of Health/National Institute of Drug Abuse is “1U18DA052402”.

The authors apologize for this error and state that this does not change the scientific conclusions of the article in any way. The original article has been updated.

